# Early C-reactive protein as a predictive biomarker for postoperative complications following robot-assisted surgery for rectal cancer

**DOI:** 10.1007/s13304-025-02379-8

**Published:** 2025-08-28

**Authors:** Fuminori Teraishi, Ryusei Takahashi, Hiroki Okabayashi, Masashi Utsumi, Hideaki Miyaso, Ryohei Shoji, Toshiyoshi Fujiwara, Toshiharu Mitsuhashi, Masaru Inagaki

**Affiliations:** 1 Department of Surgery, NHO Fukuyama Medical Center, Hiroshima, 720-8520 Japan; 2 Department of Gastroenterological Surgery, Okayama University Graduate School of Medicine, Dentistry and Pharmaceutical Sciences, Okayama, Japan; 3 Center for Innovative Clinical Medicine, Okayama University Hospital, Okayama, Japan

**Keywords:** Robot-assisted surgery, Rectal cancer, Postoperative complication, C-reactive protein

## Abstract

This retrospective cohort study aimed to assess the predictive value of early postoperative C-reactive protein (CRP) levels for complications following robot-assisted rectal surgery (RARS) for rectal cancer. We analyzed data from 117 consecutive patients who underwent elective RARS at Okayama University Hospital between September 2020 and January 2025. Serum CRP levels were routinely measured preoperatively and on postoperative days (POD) 1 and 4. The primary outcome was the occurrence of any postoperative complication within 30 days, classified according to the Clavien–Dindo grading system. Postoperative complications were observed in 26 patients, representing 22.2% of the cohort. Univariate analysis revealed that several factors were significantly associated with complications, including older age, higher ASA score, neoadjuvant therapy, stoma creation, prolonged operative time, and elevated CRP levels on POD1 and POD4. Notably, multivariate logistic regression analysis identified POD1 CRP as a robust independent predictor of overall postoperative complications (adjusted odds ratio 0.77, 95% confidence interval (CI) [0.63–0.93], *p* < 0.01). In the ROC analysis, the AUC was 0.735 (bootstrap bias-corrected 95% CI 0.544–0.848). The optimal cutoff value of POD1 CRP was 5.63 mg/dl, at which Youden’s index, yielding a sensitivity of 0.615 and specificity of 0.868. In conclusion, early postoperative measurement of CRP on POD1 serves as a valuable and independent biomarker for predicting complications following RARS for rectal cancer. Incorporating POD1 CRP into postoperative surveillance may facilitate the early identification of high-risk patients, thereby facilitating timely interventions and ultimately improving surgical outcomes in this patient population.

**Supplementary Information:**

The online version contains supplementary material available at 10.1007/s13304-025-02379-8.

## Introduction

Robot-assisted rectal surgery (RARS) has increasingly become a vital component of colorectal surgery, offering significant advantages, such as enhanced dexterity and visualization, particularly within the confined pelvic space required for rectal cancer resections [1, 2]. Systematic reviews and meta-analyses indicate that RARS may yield outcomes comparable with, or even superior to, those achieved through conventional laparoscopic or open surgical techniques across various clinical settings [1, 3]. However, large randomized controlled trials comparing robotic and laparoscopic approaches to rectal cancer surgery have produced nuanced findings regarding short-term outcomes, such as conversion rates and operative time [4, 5]. Emerging evidence suggests that RARS may be associated with a diminished postoperative surgical stress response [6]. Despite these technological advancements and the established feasibility of RARS even in advanced cases [6], postoperative complications following rectal cancer surgery remain a significant source of morbidity [7]. Therefore, the early identification of patients at a high risk of developing complications is crucial for facilitating timely intervention and improving overall outcomes. C-reactive protein (CRP), an acute-phase reactant, is a widely recognized biomarker of systemic inflammation and has been investigated as a predictor of complications, such as anastomotic leakage following colorectal surgery [8]. However, the specific predictive value of early postoperative CRP elevation, particularly on postoperative day 1, in the context of robotic rectal cancer surgery requires further investigation.

This study aims to analyze the risk factors associated with postoperative complications following RARS for rectal cancer, with a particular emphasis on assessing the predictive utility of CRP levels measured on postoperative day (POD) 1.

## Materials and methods

### Study design and patient population

This study was designed as a retrospective cohort analysis conducted at Okayama University Hospital, a tertiary referral center with extensive experience in robotic colorectal surgery. We reviewed consecutive patients who underwent elective robot-assisted surgery for primary rectal cancer between September 2020 and January 2025. Patients who required emergency surgery, palliative procedures, or those with incomplete perioperative data were excluded from the analysis. The study protocol received approval from the Okayama University Hospital Review Board (approval No. 2004-028), and the requirement for informed consent was waived owing to the retrospective nature of the analysis.

### Surgical procedure of robot-assisted rectal surgery (RARS)

All procedures were performed by an experienced colorectal surgeon (F. Teraishi, who is credentialed in robot-assisted surgery using the da Vinci Surgical System (Intuitive Surgical Inc., Sunnyvale, CA, USA). All procedures were conducted under general anesthesia, with patients positioned in the lithotomy stance. The surgical technique was robot-assisted total mesorectal excision [9, 10]. The details of the surgery are described in the Appendix. Perioperative care adhered to an established perioperative management center protocol implemented at the Okayama University Hospital.

### Data collection and definitions

Demographic data including age, sex, and body mass index (BMI), as well as comorbidities assessed by the American Society of Anesthesiologists (ASA) score, tumor characteristics (such as stage, distance from the anal verge, neoadjuvant therapy), intraoperative variables (including operative time, estimated blood loss, conversion to open or laparoscopic surgery, type of anastomosis, and creation of diverting stoma), and postoperative outcomes were extracted from a prospectively maintained institutional database and electronic health records.

Serum CRP levels were routinely measured preoperatively and on POD1 (16 ± 4 h) and POD4 as part of our standard postoperative monitoring protocol. The primary outcome measure was the occurrence of any postoperative complication occurring within 30 days of surgery, graded according to the Clavien–Dindo (C–D) classification [11]. Major complications were defined as C–D grade ≥ IIIb, necessitating surgical, endoscopic, or radiological intervention under general anesthesia. Anastomotic leakage (AL) was defined in accordance with the criteria established by the International Study Group of Rectal Cancer [12]. Surgical site infections were defined based on the guidelines set forth by the Centers for Disease Control and Prevention [13].

### Statistical analysis

Continuous variables were reported as medians with interquartile ranges (IQRs), whereas categorical variables were expressed as counts and percentages (%). To compare patients with postoperative complications (C–D all grade) to those without, we employed the independent samples *t*-test or Mann–Whitney U test for continuous variables, and the Chi-square or Fisher's exact test for categorical variables.

In the univariate analysis, variables demonstrating a potential association with complications (*p* < 0.05), along with clinically relevant factors, such as age, BMI, ASA score, neoadjuvant therapy, operative time, stoma creation, POD1 CRP, were included in a multivariate logistic regression model. This model utilized a backward stepwise selection method to identify the independent predictors of overall postoperative complications. In the primary model and multivariate analyses, POD1 CRP was treated as a continuous variable. Odds ratios (ORs) with 95% confidence intervals (CIs) were calculated. To determine the optimal cutoff value of POD1 CRP for predicting complications, ROC curve analysis was performed using five-fold cross-validation, considering the sample size. The resulting five ROC curves were aggregated using lowess smoothing. The mean AUC and its 95% bootstrap bias-corrected confidence interval were calculated based on 1000 bootstrap resamples. The optimal cutoff point was then selected to maximize Youden’s index on the integrated data [14]. A p-value of less than 0.05 was deemed statistically significant. All statistical analyses were performed using GraphPad Prism 6 (GraphPad Software, Boston, MA, USA) or SPSS Statistics version 25.0 (IBM Corp., Armonk, NY, USA).

## Results

### Patient characteristics and surgical findings

The patient characteristics are listed in Table 1. In total, 117 patients who underwent robot-assisted surgery for rectal cancer during the study period satisfied the inclusion criteria. The mean age of the participants was 66, with 59.0% being male. The median BMI was recorded at 22.4 kg/m^2^. Most patients were classified as ASA II or III, accounting for 76 patients (64.1%). The median distance from the anal verge to the lower tumor margin was 9 cm. Pathological diagnoses included rectal cancer in 109 cases, anal cancer in 3, neuroendocrine tumor in 3, gastrointestinal stromal tumor in 1, and melanoma in 1. Preoperative treatment was administered to 25 patients (21.4%).

**Table 1:** Baseline characteristics of 118 rectal neoplasm patients undergoing RARS

Age, years, median (IQR)	66 (54–73)
Gender, n (%)	
Male	69 (59.0)
Female	48 (41.0)
BMI, kg/m^2^, median (IQR)	22.4 (20.3–26.0)
ASA classification, n (%)	
1	42 (35.9)
2/3	75 (64.1)
Hemoglobin, g/dl, median (IQR)	13.7 (12.4–15.0)
CEA, ng/ml, median (IQR)	2.8 (1.9–5.2)
CA19-9, ng/ml, median (IQR)	14.5 (7.8–28.7)
Tumor location, n (%)	
Rectosigmoid	35 (29.9)
Upper rectum	38 (32.5)
Lower rectum	44 (37.6)
Distance from AV, cm, median (IQR)	9.0 (5.0–15.0)
Preoperative CRP, mg/dl, median (IQR)	0.1 (0–0.2)
Neoadjuvant therapy, n (%)	25 (21.4)
pStage, n (%)	
0	4
I	36
II	24
III	36
IV	9
Others	8

*ASA* American Society of Anesthesiologists, *AV* anal verge, *BMI* body mass index, *CA19-9* carbohydrate antigen, *CEA* carcinoembryonic antigen, *IQR* interquartile range, *pStage* pathological stage

The surgical findings are listed in Table 2. The most frequently performed procedure was low anterior resection, which was conducted in 77 cases (65.8%). A diverting stoma was created in 49 cases (41.9%). D3 lymph node dissection was performed in 93 cases (79.5%), whereas lateral lymph node dissection was performed in 11 cases (9.4%). Notably, conversions to open surgery were not required. The median operative and console times were 337 and 233 min, respectively, and the median intraoperative blood loss was 5 ml.

**Table 2:** Surgical findings of patients

Surgical procedure	
LAR	77 (65.8)
HAR	20 (17.1)
APR	17 (14.5)
ISR	2 (1.7)
PE	1 (0.9)
Stoma creation, n (%)	49 (41.9)
Lymphnode dissection	
D3	93 (79.5)
Non-D3	24 (21.5)
Lateral lymphnode dissection	11 (9.4)
Conversion, n (%)	0 (0)
Operation time, min, median (IQR)	337 (294–421)
Console time, min, median (IQR)	233 (189–289)
Blood loss, ml, median (IQR)	5 (5–50)

*APR* abdomino-perineal resection, *HAR* high anterior resection, *ISR* intersphincteric resection, *LAR* low anterior resection, *PE* pelvic exenteration

### Postoperative outcomes

The postoperative outcomes are listed in Table 3. The median CRP levels on POD1 and POD4 were 3.9 and 2.9 mg/dl, respectively. Postoperative complications were observed in 26 patients (22.2%), including ileus (10 cases), intra-abdominal abscess and anastomotic leakage (seven cases), lymphorrhea (two cases), and surgical site infection, urinary dysfunction, and deep vein thrombosis (one case each). Severe complications (C–D Classification ≥ IIIb) were observed in four patients (3.4%), with reoperation required in three instances. No mortality occurred within 30 days, and the median postoperative hospital stay was nine days.

**Table 3:** Postoperative outcomes of patients

Postoperative CRP, mg/dl, median (IQR)	
POD1	3.9 (2.4–5.4)
POD4	2.9 (1.3–6.3)
Postoperative complications	
C–D Grade I–V, n (%)	26 (22.2)
Ileus	10 (8.5)
Intra-pelvic abscess	7 (6.0)
Anastomotic leakage	7 (6.0)
Lymphorrhea	2 (1.7)
Wound infection	1 (0.9)
Neurogenic bladder	1 (0.9)
DVT	1 (0.9)
C–D Grade ≧ II, n (%)	20 (17.1)
C–D Grade ≧ IIIb, n (%)	4 (3.4)
Anastomotic leakage, n (%)	7 (6.0)
Reoperation, n (%)	3 (2.6)
30-day mortality, n (%)	0 (0)
Length of stay, days, median (IQR)	9 (8–12)

*C − D* Clavien–Dindo classification, *DVT* deep venous thrombosis, *POD* postoperative day

### Correlation with postoperative complication

An analysis of factors associated with postoperative complications is provided in Table 4. Patients who developed postoperative complications were significantly older (*p* < 0.01) and were more frequently classified as ASA class 2/3 (*p* = 0.01). Furthermore, these patients were more likely to have received preoperative treatment (*p* = 0.02). Stoma creation was more prevalent in this group (*p* < 0.001). These patients also had significantly longer operative times (*p* < 0.001) and elevated CRP levels on POD1 and POD4 (*p* < 0.001). The presence of postoperative complications was associated with significantly prolonged hospital stay (*p* < 0.001).

**Table 4:** Risk factors associated with postoperative complication

	Postoperative complication	No postoperative complication	*p* value
	N = 26	N = 91	
Age, years, median (IQR)	71 (61–76)	64 (52–71)	**< 0.01**
Gender, n (%)			0.45
Male	17 (65.4)	52 (57.1)	
Female	9 (34.6)	39 (42.9)	
BMI, kg/m^2^, median (IQR)	23.8 (20.5–26.0)	22.2 (20.2–26.2)	0.51
ASA classification, n (%)			**0.01**
1	4 (15.4)	38 (41.8)	
2/3	22 (84.6)	53 (58.2)	
Hemoglobin, g/dl, median (IQR)	13.2 (11.5–14.2)	13.8 (12.8–14.9)	0.10
Distance from AV, cm, median (IQR)	5.0 (2.0–10.0)	9.0 (5.0–15.0)	0.06
Neoadjuvant therapy, n (%)	10 (38.5)	15 (16.5)	**0.02**
Stoma creation, n (%)	20 (77)	29 (31.9)	**< 0.001**
Lymphnode dissection			0.20
D3	23 (88.5)	70 (76.9)	
Non-D3	3 (11.5)	21 (23.1)	
Lateral lymphnode dissection	5 (19.2)	6 (6.6)	0.052
Operation time, min, median (IQR)	431 (335–507)	324 (286–408)	**< 0.001**
Blood loss, ml, median (IQR)	8 (5–120)	5 (5–50)	0.31
Postoperative CRP, mg/dl, median (IQR)			
POD1	6.2 (3.3–8.8)	3.6 (2.2–5.1)	**< 0.001**
POD4	8.5 (5.4–12.2)	2.1 (1.2–4.1)	**< 0.001**
Length of stay, days, median (IQR)	16 (14–27)	9 (8–10)	**< 0.001**

Bold values indicate statistical significance (*p* < 0.05)

*C–D* Clavien–Dindo, *DVT* deep venous thrombosis, *POD* postoperative day

**Fig. 1:**
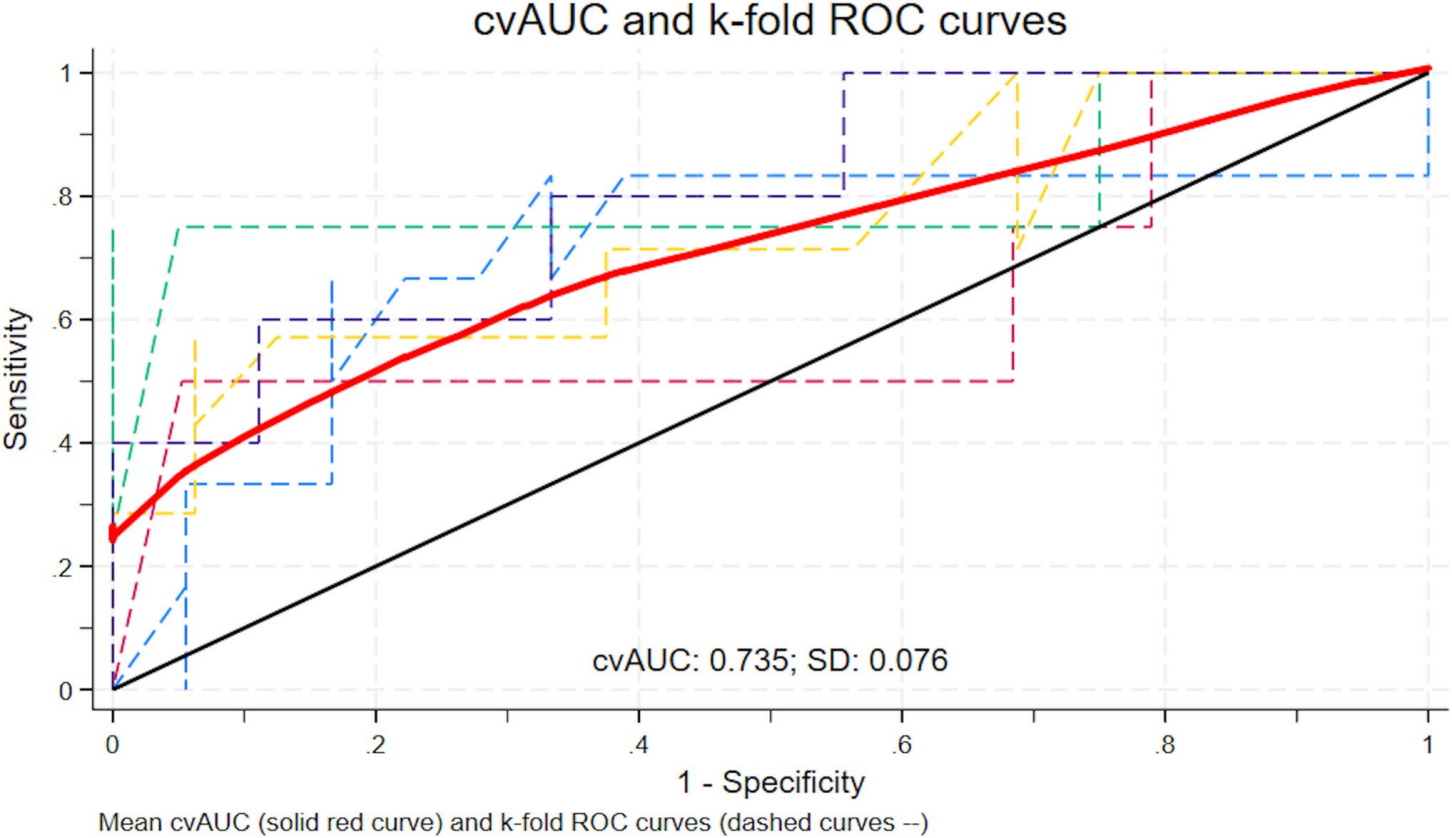
Receiver operating characteristic curve based on five-fold cross-validation. Dashed lines represent the Receiver Operating Characteristic curves obtained from each of the five validation folds. The solid red line represents the lowess-smoothed average of these curves. The cross-validated area under the curve (cvAUC) was 0.735, with a standard deviation of 0.076 (color figure online)

The findings from the multivariate logistic regression analysis, which incorporated these variables are listed in Table 5. After adjusting for confounding factors, POD1 CRP emerged as a strong and independent predictor of overall postoperative complications (adjusted odds ratio 0.77, 95% confidence interval (CI) [0.63–0.93], *p* < 0.01). In the ROC analysis with five-fold cross-validation, the AUC was 0.735 (bootstrap bias-corrected 95% CI: 0.544–0.848) (Fig. 1). The optimal cutoff value of POD1 CRP was 5.63 mg/dl, at which Youden’s index reached its maximum (0.484), yielding a sensitivity of 0.615 and specificity of 0.868 (Suppl Table).

**Table 5:** Results of multivariate analysis on factors related to postoperative complications

Variable	Multivariable model	
	OR (95% CI)	*p* value
Age ≧ 70 years	1.87 (0.64–5.44)	0.25
Distance from AV ≦ 5 cm	1.46 (0.37–5.78)	0.59
ASA 1	0.38 (0.10–1.43)	0.15
No neoadjuvant therapy	0.52 (0.16–1.73)	0.29
Operation time ≧ 420 min	2.58 (0.83–8.08)	0.10
Stoma creation	1.77 (0.35–8.82)	0.49
POD1 CRP	0.77 (0.63–0.93)	**< 0.01**

Bold values indicate statistical significance (*p* < 0.05)

*ASA* American Society of Anesthesiologists, *AV* anal verge, *CI* confidence interval, *POD* postoperative day

## Discussion

This study investigated the risk factors associated with complications following robot-assisted rectal cancer surgery, with a particular emphasis on the predictive value of early postoperative CRP levels. Our key finding, derived from the multivariate analysis, indicates that serum CRP levels measured on POD1 serve as a robust and independent predictor of overall postoperative complications. This finding not only corroborates but also expands upon previous research that underscores the importance of CRP monitoring following colorectal surgery [15–17]. Notably, our study validates the strong predictive capacity of CRP at a very early time point (POD1) within the specific context of robotic rectal resection.

The postoperative inflammatory response, as indicated by biomarkers, such as CRP, is a known consequence of surgical trauma [18]. While some studies suggest that robot-assisted surgery may mitigate the systemic inflammatory response compared with laparoscopic or open approaches, potentially owing to reduced tissue handling or smaller incisions [6, 18, 19], significant inflammation still occurs. Our findings suggest that even within the potentially modulated inflammatory environment associated with RAS, the magnitude of CRP elevation on POD1 remains a critical indicator of the patient's subsequent clinical course. An exaggerated early CRP response may greater surgical stress, occult tissue injury, or the incipient stages of an infectious process or anastomotic compromise [20, 21].

Previous studies have explored CRP as an early warning marker, often concentrating on values measured between POD3 and 5 or analyzing CRP trajectories [17, 22]. While CRP kinetics provide valuable insights, utilizing a POD1 CRP value presents a distinct clinical advantage by facilitating the earlier identification of high-risk patients. Several studies have reported varying CRP cut-off values—typically exceeding 10–15 mg/dl on POD3-5—for predicting complications, particularly AL [15, 16, 22, 23]. Our research identified an optimal cutoff on POD1 of 5.63 mg/dl, establishing a specific and actionable threshold within the context of robot-assisted surgery. Patients exceeding this value on the first postoperative day should be monitored more closely. This early stratification could inform clinical decisions, such as delaying oral intake, intensifying monitoring protocols, or initiating earlier diagnostic imaging if clinical suspicion arises. Such proactive measures may help mitigate the severity of complications, such as AL [8, 23, 24]. Conversely, a low CRP level on POD1 may provide reassurance and support adherence to the ERAS pathways.

The non-specific nature of CRP must be acknowledged; elevated levels indicate inflammation but do not specify its precise cause [16]. However, in the context of recent major pelvic surgery, a significantly elevated CRP level on POD1 strongly suggests a deviation from the anticipated uncomplicated recovery trajectory. Its predictive value is further enhanced when considered alongside clinical assessments and potentially other biomarkers or preoperative risk scores [25, 26]. Although the POD1 CRP value may be influenced by neoadjuvant treatment and comorbidities, there was no difference in the POD1 CRP value between patients with and without systemic comorbidity (data not shown). As for neoadjuvant treatment, POD1 CRP values were significantly higher in the neoadjuvant treatment group (*p* = 0.02) (data not shown), and the possibility of confounding cannot be ruled out. Our multivariate analysis validates the independent predictive power, even after accounting for established risk factors, such as male sex and operative duration, both of which were also significant in our cohort, aligning with the existing literature.

This study leverages the advantages of a homogeneous surgical platform—robot-assisted surgery—conducted within a high-volume center that employs standardized techniques and perioperative protocols. This approach minimizes variability associated with surgical methods. The application of robust multivariate analysis enhances the validity of our findings, particularly in identifying POD1 CRP as an independent predictor of outcomes. However, the limitations inherent to its retrospective design include a potential selection bias and reliance on accurately recorded data. While we adjusted for several confounders, residual confounding factors could not be entirely excluded. Additionally, our findings are derived from a single institution, potentially limiting generalizability; therefore, multicenter validation is essential. Furthermore, the number of postoperative complications in this study was small (26), so the multivariate model may be underpowered. We focused primarily on overall complications; although AL demonstrated similar trends, larger cohorts may be necessary to establish POD1 CRP thresholds specifically for AL prediction following robot-assisted surgery [24]. Furthermore, the impact of preoperative factors, such as nutritional status or systemic inflammation—potentially assessed through tools, such as the Naples prognostic score [26]—on postoperative CRP response merits further investigation.

## Conclusion

This study demonstrated that serum C-reactive protein levels measured on the first postoperative day serve as a readily accessible, inexpensive, and robust independent predictor of subsequent complications following robot-assisted rectal cancer surgery. Incorporating POD1 CRP assessments into standard postoperative surveillance protocols can enhance early risk stratification, potentially facilitating timely interventions and optimizing patient management in the context of advanced minimally invasive colorectal surgery.

Future research should aim to prospectively validate the POD1 CRP threshold identified in diverse robot-assisted surgery populations. Additionally exploring the combination of POD1 CRP with other early biomarkers, such as procalcitonin, or analysis of drain fluid [21], could significantly enhance the predictive accuracy. Importantly, studies that evaluate whether interventions guided by early CRP levels can actively improve patient outcomes are critically required.

## Supplementary Information

Below is the link to the electronic supplementary material.

Supplementary file1 (DOCX 16 KB)

Supplementary file2 (DOCX 34 KB)

## Data Availability

The data supporting the findings of this study are available from the corresponding author upon reasonable request.
