# Supplementary material for: Early C-reactive protein as a predictive biomarker for postoperative complications following robot-assisted surgery for rectal cancer
Source: Updates Surg. 2025 Aug 28;78(2):611–7. doi: 10.1007/s13304-025-02379-8 (PMC13212727; doi:10.1007/s13304-025-02379-8)
Supplement: Supplementary file 1 — Supplementary file1 (DOCX 16 KB) [file 13304_2025_2379_MOESM1_ESM.docx]

**Surgical details**

The surgical technique employed a 5-trocar approach, which is standard for robotic total mesorectal excision [1, 2]. This technique included high ligation of the inferior mesenteric artery and vein, complete mobilization of the rectum within the mesorectal fascia, and preservation of the surrounding nerves. The type of anastomosis performed—either colorectal or coloanal—was determined based on tumor height and patient factors.

**References**

1. Heald RJ, Ryall RD (1986) Recurrence and survival after total mesorectal excision for rectal cancer. Lancet 1(8496):1479-82
2. Crolla R, Coffey JC, Consten EJC (2022) The Mesentery in Robot-Assisted Total Mesorectal Excision. Clin Colon Rectal Surg 35(4):298-305
