# Supplementary material for: Early C-reactive protein as a predictive biomarker for postoperative complications following robot-assisted surgery for rectal cancer
Source: Updates Surg. 2025 Aug 28;78(2):611–7. doi: 10.1007/s13304-025-02379-8 (PMC13212727; doi:10.1007/s13304-025-02379-8)
Supplement: Supplementary file 2 — Supplementary file2 (DOCX 34 KB) [file 13304_2025_2379_MOESM2_ESM.docx]

**Supplementary Table**

The optimal cutoff value of POD1 CRP was 5.63 mg/dl, at which Youden’s index reached its maximum (0.484), yielding a sensitivity of 0.615 and specificity of 0.868 (highlighted in yellow).

| Predicted probability | POD1 CRP | Sensitivity | Specificity | Youden's Index |
| --- | --- | --- | --- | --- |
| 0 | infinite negative | 1.000 | 0.000 | 0.000 |
| 0.04 | -0.39 | 0.962 | 0.000 | -0.039 |
| 0.05 | 0.24 | 0.962 | 0.017 | -0.022 |
| 0.06 | 0.76 | 0.962 | 0.044 | 0.006 |
| 0.07 | 1.2 | 0.955 | 0.092 | 0.047 |
| 0.08 | 1.59 | 0.923 | 0.159 | 0.082 |
| 0.09 | 1.94 | 0.915 | 0.229 | 0.144 |
| 0.1 | 2.25 | 0.840 | 0.267 | 0.107 |
| 0.12 | 2.8 | 0.779 | 0.345 | 0.124 |
| 0.13 | 3.05 | 0.769 | 0.407 | 0.176 |
| 0.14 | 3.28 | 0.769 | 0.445 | 0.214 |
| 0.15 | 3.5 | 0.762 | 0.492 | 0.254 |
| 0.16 | 3.7 | 0.692 | 0.517 | 0.209 |
| 0.17 | 3.9 | 0.692 | 0.555 | 0.247 |
| 0.18 | 4.08 | 0.667 | 0.586 | 0.253 |
| 0.19 | 4.26 | 0.654 | 0.615 | 0.269 |
| 0.2 | 4.44 | 0.654 | 0.648 | 0.302 |
| 0.21 | 4.6 | 0.654 | 0.676 | 0.330 |
| 0.22 | 4.76 | 0.654 | 0.703 | 0.357 |
| 0.23 | 4.92 | 0.654 | 0.736 | 0.390 |
| 0.24 | 5.07 | 0.654 | 0.775 | 0.429 |
| 0.25 | 5.21 | 0.615 | 0.797 | 0.412 |
| 0.26 | 5.35 | 0.615 | 0.828 | 0.443 |
| **0.28** | **5.63** | **0.615** | **0.868** | **0.484** |
| 0.29 | 5.76 | 0.558 | 0.879 | 0.437 |
| 0.3 | 5.89 | 0.487 | 0.886 | 0.374 |
| 0.32 | 6.14 | 0.462 | 0.901 | 0.363 |
| 0.33 | 6.26 | 0.423 | 0.912 | 0.335 |
| 0.34 | 6.38 | 0.423 | 0.934 | 0.357 |
| 0.35 | 6.5 | 0.385 | 0.934 | 0.319 |
| 0.36 | 6.62 | 0.385 | 0.945 | 0.330 |
| 0.38 | 6.85 | 0.346 | 0.956 | 0.302 |
| 0.4 | 7.08 | 0.308 | 0.956 | 0.264 |
| 0.43 | 7.41 | 0.308 | 0.967 | 0.275 |
| 0.44 | 7.52 | 0.269 | 0.967 | 0.236 |
| 0.45 | 7.63 | 0.269 | 0.978 | 0.247 |
| 0.56 | 8.82 | 0.231 | 0.978 | 0.209 |
| 0.57 | 8.93 | 0.192 | 0.978 | 0.170 |
| 0.6 | 9.27 | 0.192 | 0.989 | 0.181 |
| 0.62 | 9.49 | 0.154 | 0.989 | 0.143 |
| 0.77 | 11.43 | 0.154 | 1.000 | 0.154 |
| 0.8 | 11.91 | 0.096 | 1.000 | 0.096 |
| 0.86 | 13.07 | 0.039 | 1.000 | 0.039 |
| 0.9 | 14.1 | 0.000 | 1.000 | 0.000 |
